# Supplementary material for: PGRMC1 phosphorylation affects cell shape, motility, glycolysis, mitochondrial form and function, and tumor growth
Source: BMC Mol Cell Biol. 2020 Apr 3;21:24. doi: 10.1186/s12860-020-00256-3 (PMC7119165; doi:10.1186/s12860-020-00256-3)
Supplement: Supplementary file 1 — Additional file 1:Figure S1. Detailed views of selected pathways identified by WebGestalt analyses. Related to Fig. 3. All panels are adapted from File S6. Heat map colors follow Fig. 3. (A) Principal component (PC) analysis of SWATH-MS proteomics results showing distribution of PC1 and PC2. PC1 corresponded to pathways associated with ribosomes and translation, while PC2 corresponded to pathways associated with mRNA splicing processing (see File S3). (B) Proteins associated with PI3K/AKT activity (WebGestalt Database: PC, DB_ID:1648, “Class I PI3K signaling events mediated by AKT”) are less abundant in TM cells. (C) F1/F0 ATPase subunits elevated in WT and TM cells. (D) Abundances of proteins associated with protein folding and microtubule function are altered by PGRMC1 phosphorylation status. Proteins detected in any of the following WebGestalt pathways or functions (1–4) or a manual search (5) are mapped against their expression profiles. 1) cellular component chaperonin-containing T-complex GO:0005832. 2) PC pathway Chaperonin-mediated protein folding DB_ID:710. 3) cellular component microtubule GO:0005874. 4) PC pathway Protein folding DB_ID:712. 5) Description from the list of 243 proteins (File S6) contains keywords “tubulin” or “microtubule” (manual search) (Adapted from File S6). 2-tailed t-test p-values for all sample comparisons are available in File S4. (E) Proteins associated with nuclear import/export that are elevated in DM cells. (F) Antigen processing and presentation enzymes are affected by PGRMC1 phosphorylation status. Manual additions to KEGG pathway ID:04612 “Antigen processing and presentation" (no yellow shading: from File S6 and File S5) are indicated with yellow highlighting. Figure S2. Highest and lowest differentially abundant proteins. Panels show the six most (+) and least (−) abundant proteins for each cell type that were significantly differentially abundant between cell types. Related to Fig. 3. Identical colored symbols depict the s [file 12860_2020_256_MOESM1_ESM.pdf]

FIGURES

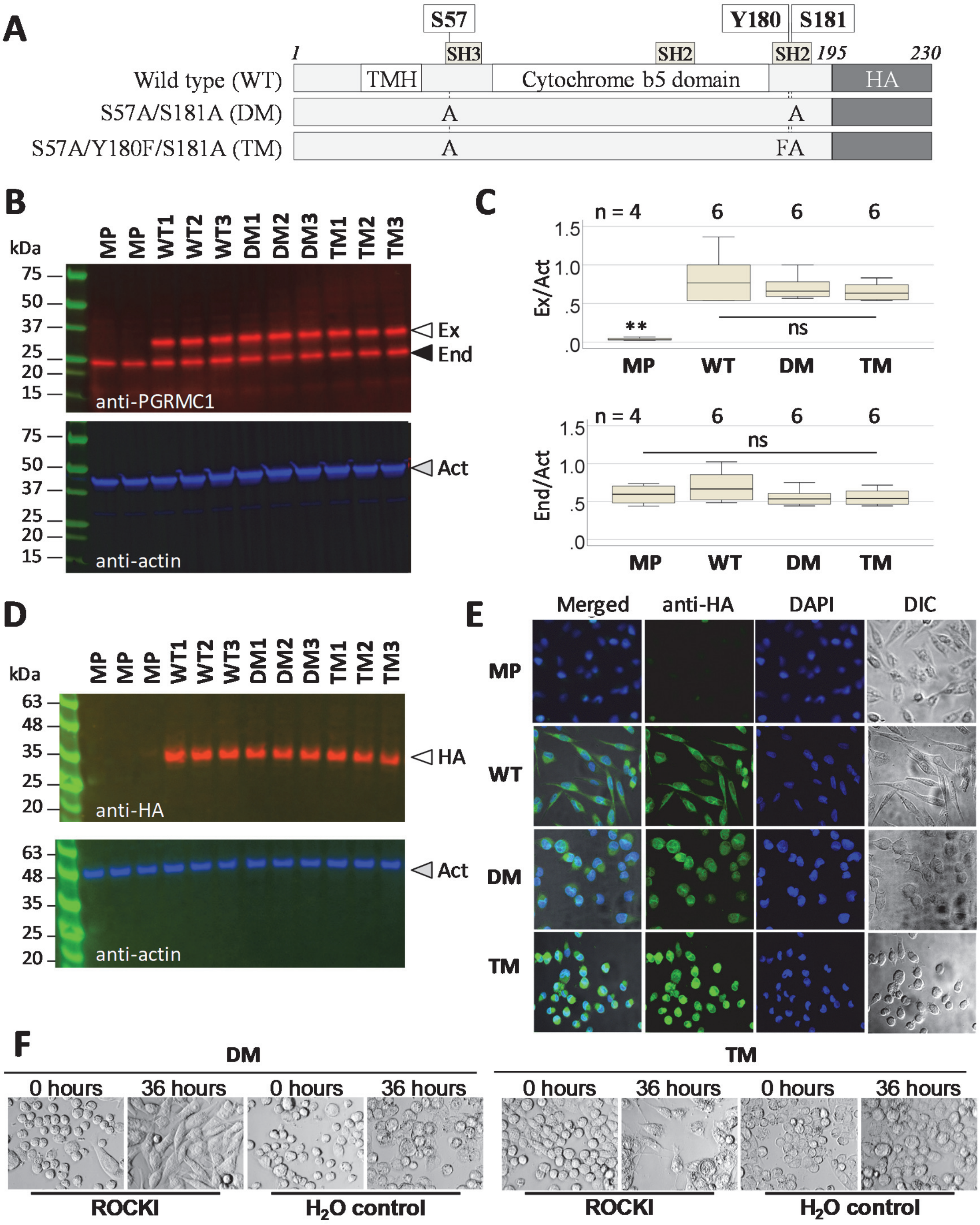

Fig. 1

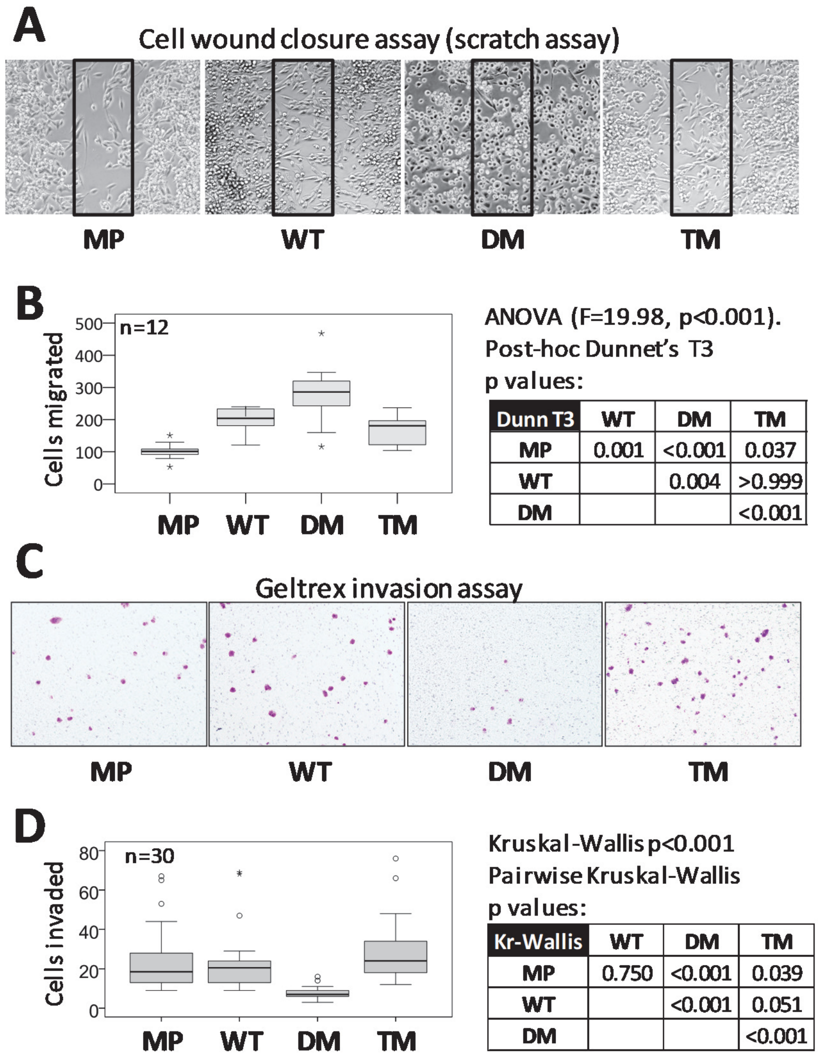

Dunn T3	WT	DM	TM
MP	0.001	<0.001	0.037
WT		0.004	>0.999
DM			<0.001

Kr-Wallis	WT	DM	TM
MP	0.750	<0.001	0.039
WT		<0.001	0.051
DM			<0.001

Fig. 2.

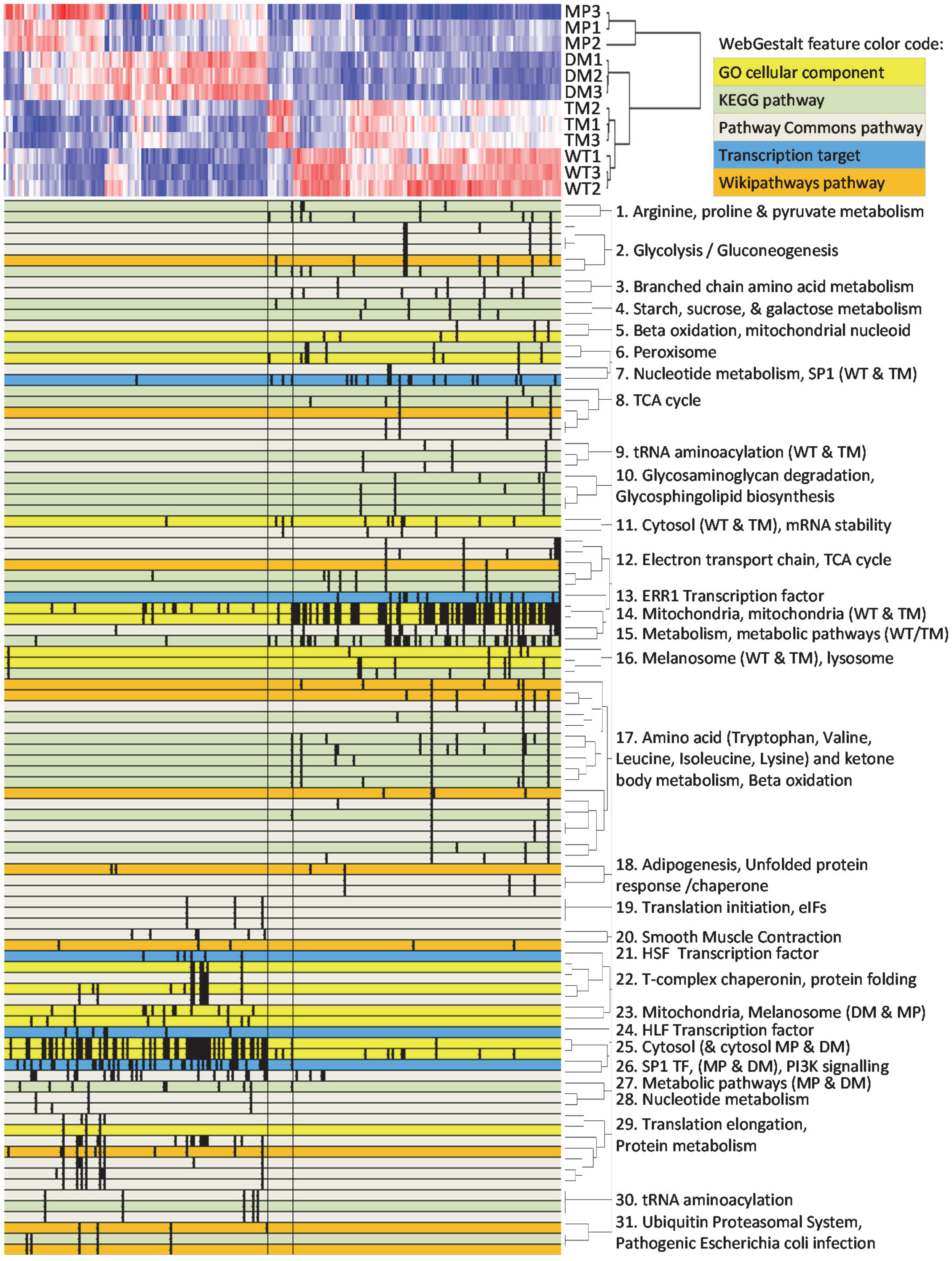

MP3
MP1
MP2
DM1
DM2
DM3
TM2
TM1
TM3
WT1
WT3
WT2
WebGestalt feature color code:
GO cellular component
KEGG pathway
Pathway Commons pathway
Transcription target
Wikipathways pathway
1. Arginine, proline & pyruvate metabolism
2. Glycolysis / Gluconeogenesis
3. Branched chain amino acid metabolism
4. Starch, sucrose, & galactose metabolism
5. Beta oxidation, mitochondrial nucleoid
6. Peroxisome
7. Nucleotide metabolism, SP1 (WT & TM)
8. TCA cycle
9. tRNA aminoacylation (WT & TM)
10. Glycosaminoglycan degradation, Glycosphingolipid biosynthesis
11. Cytosol (WT & TM), mRNA stability
12. Electron transport chain, TCA cycle
13. ERR1 Transcription factor
14. Mitochondria, mitochondria (WT & TM)
15. Metabolism, metabolic pathways (WT/TM)
16. Melanosome (WT & TM), lysosome
17. Amino acid (Tryptophan, Valine, Leucine, Isoleucine, Lysine) and ketone body metabolism, Beta oxidation
18. Adipogenesis, Unfolded protein response /chaperone
19. Translation initiation, eIFs
20. Smooth Muscle Contraction
21. HSF Transcription factor
22. T-complex chaperonin, protein folding
23. Mitochondria, Melanosome (DM & MP)
24. HLF Transcription factor
25. Cytosol (& cytosol MP & DM)
26. SP1 TF, (MP & DM), PI3K signalling
27. Metabolic pathways (MP & DM)
28. Nucleotide metabolism
29. Translation elongation, Protein metabolism
30. tRNA aminoacylation
31. Ubiquitin Proteasomal System, Pathogenic Escherichia coli infection

Fig. 3.

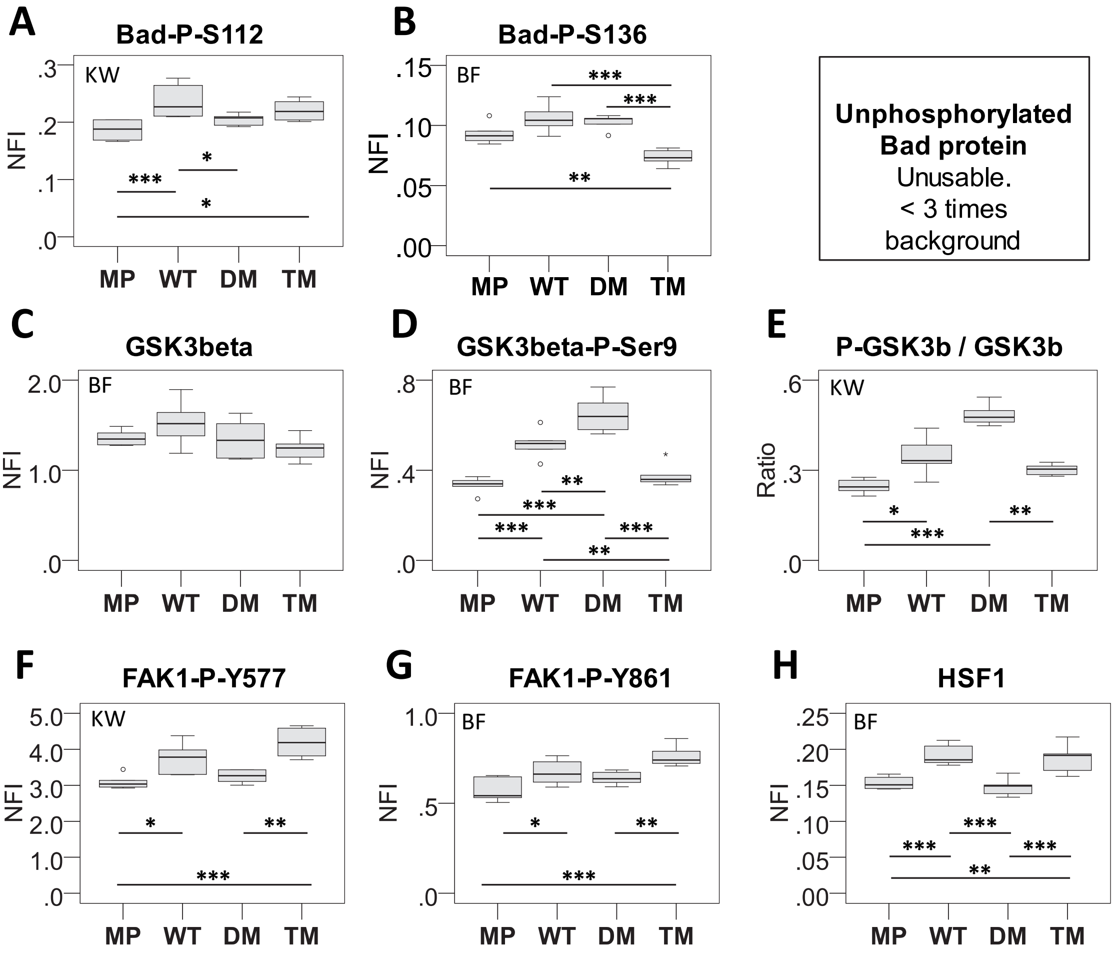
A
Bad-P-S112
KW
NFI
.3
.2
.1
.0
***
*
*
MP WT DM TM
B
Bad-P-S136
BF
NFI
.15
.10
.05
.00
***
***
**
MP WT DM TM
Unphosphorylated
Bad protein
Unusable.
< 3 times
background
C
GSK3beta
BF
NFI
2.0
1.0
.0
MP WT DM TM
D
GSK3beta-P-Ser9
BF
NFI
.8
.4
.0
**
***
***
***
**
MP WT DM TM
E
P-GSK3b / GSK3b
KW
Ratio
.6
.3
.0
*
***
**
MP WT DM TM
F
FAK1-P-Y577
KW
NFI
5.0
4.0
3.0
2.0
1.0
.0
*
**
***
MP WT DM TM
G
FAK1-P-Y861
BF
NFI
1.0
.5
.0
*
**
***
MP WT DM TM
H
HSF1
BF
NFI
.25
.20
.15
.10
.05
.00
***
***
***
**
MP WT DM TM

Fig. 4.

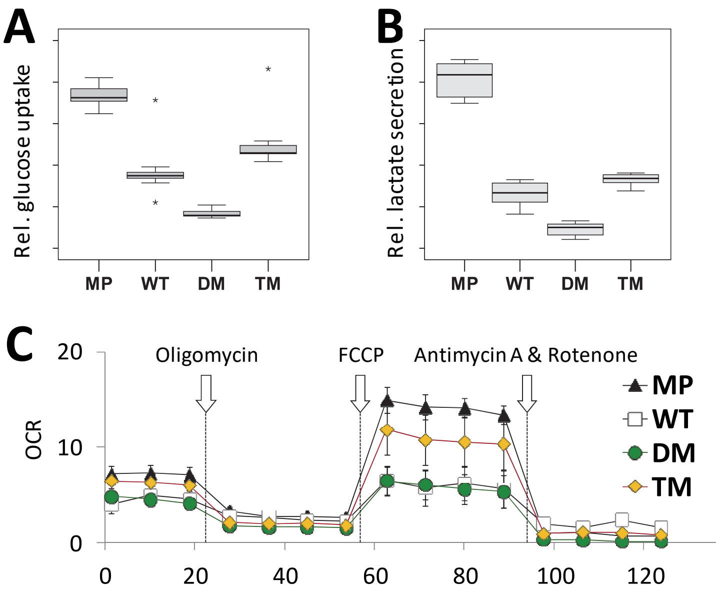
A
Rel. glucose uptake
MP
WT
DM
TM
B
Rel. lactate secretion
MP
WT
DM
TM
C
OCR
Oligomycin
FCCP
Antimycin A & Rotenone
MP
WT
DM
TM
0
10
20
0
20
40
60
80
100
120

Fig. 5.

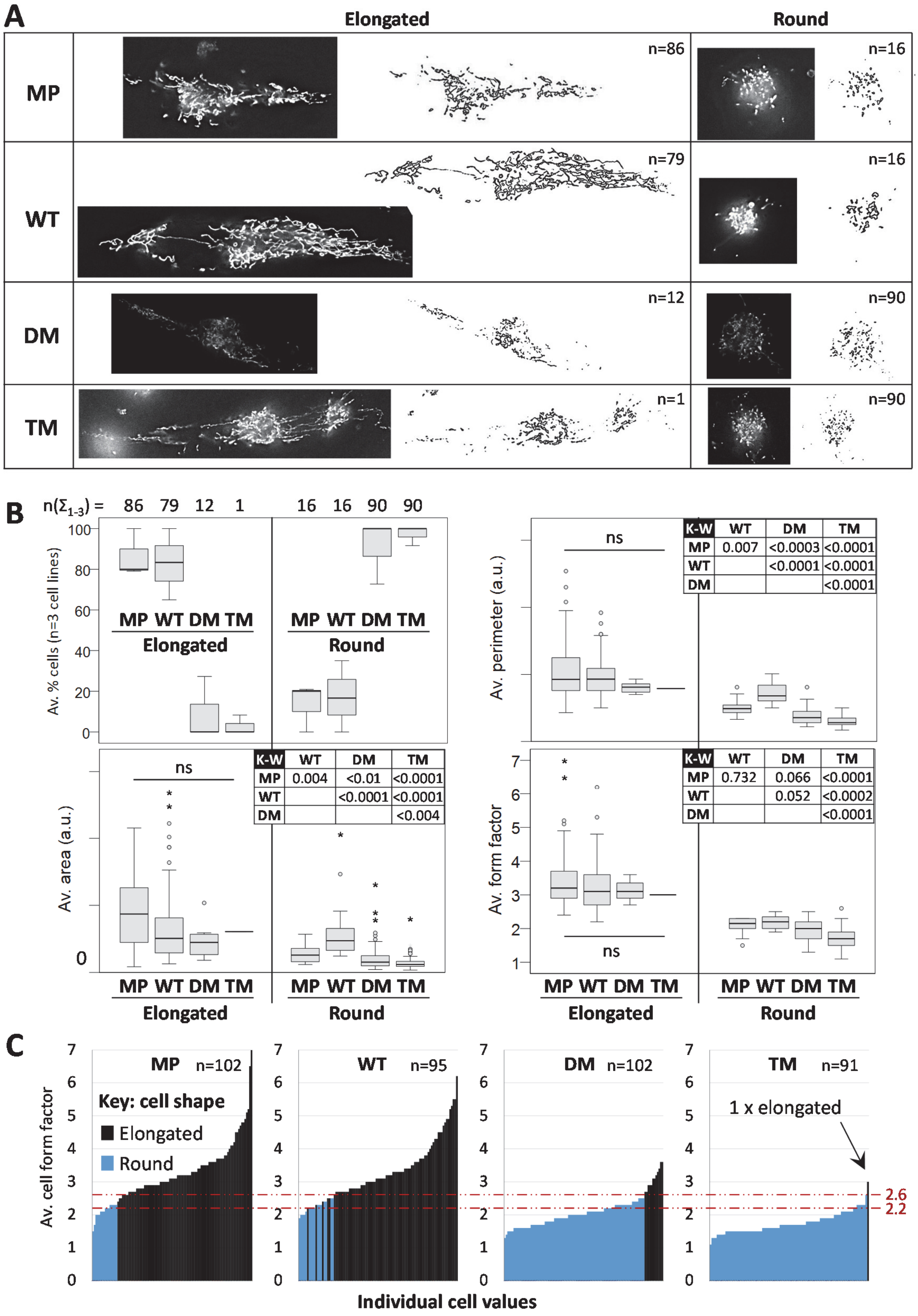
A
Elongated
Round
MP
WT
DM
TM
n=86
n=16
n=79
n=16
n=12
n=90
n=1
n=90
B
n(Σ1-3) = 86 79 12 1 16 16 90 90
Av. % cells (n=3 cell lines)
MP WT DM TM
Elongated
MP WT DM TM
Round
Av. perimeter (a.u.)
ns
K-W WT DM TM
MP 0.007 <0.0003 <0.0001
WT <0.0001 <0.0001
DM <0.0001
Av. area (a.u.)
ns
K-W WT DM TM
MP 0.004 <0.01 <0.0001
WT <0.0001 <0.0001
DM <0.004
Av. form factor
ns
K-W WT DM TM
MP 0.732 0.066 <0.0001
WT 0.052 <0.0002
DM <0.0001
C
Av. cell form factor
MP n=102
WT n=95
DM n=102
TM n=91
Key: cell shape
Elongated
Round
1 x elongated
2.6
2.2
Individual cell values

Fig. 6.

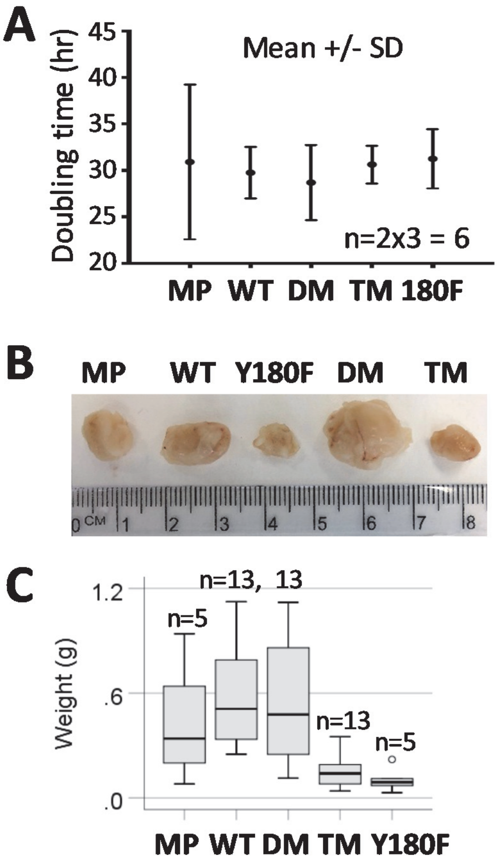

(C) ANOVA (F>7.32, p<0.0005)
Post-hoc Dunnet's T3 p values:

C	WT	DM	TM	Y180F
MP	0.992	0.993	0.564	0.455
WT		>.999	<0.0011	<0.0005
DM			<0.010	<0.005
TM				0.948

Fig. 7.

SUPPORTING INFORMATION FIGURES

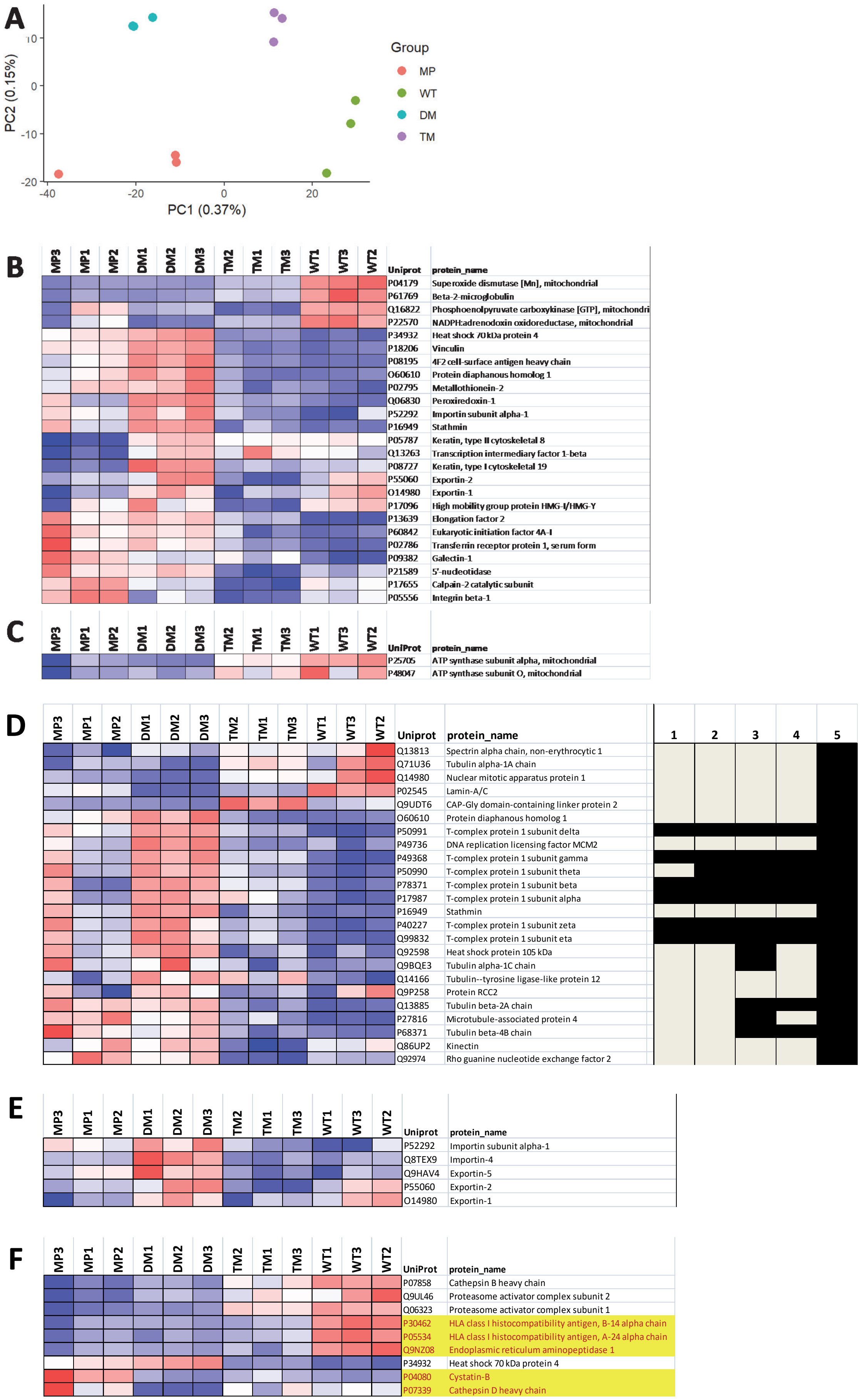

Fig. S1.

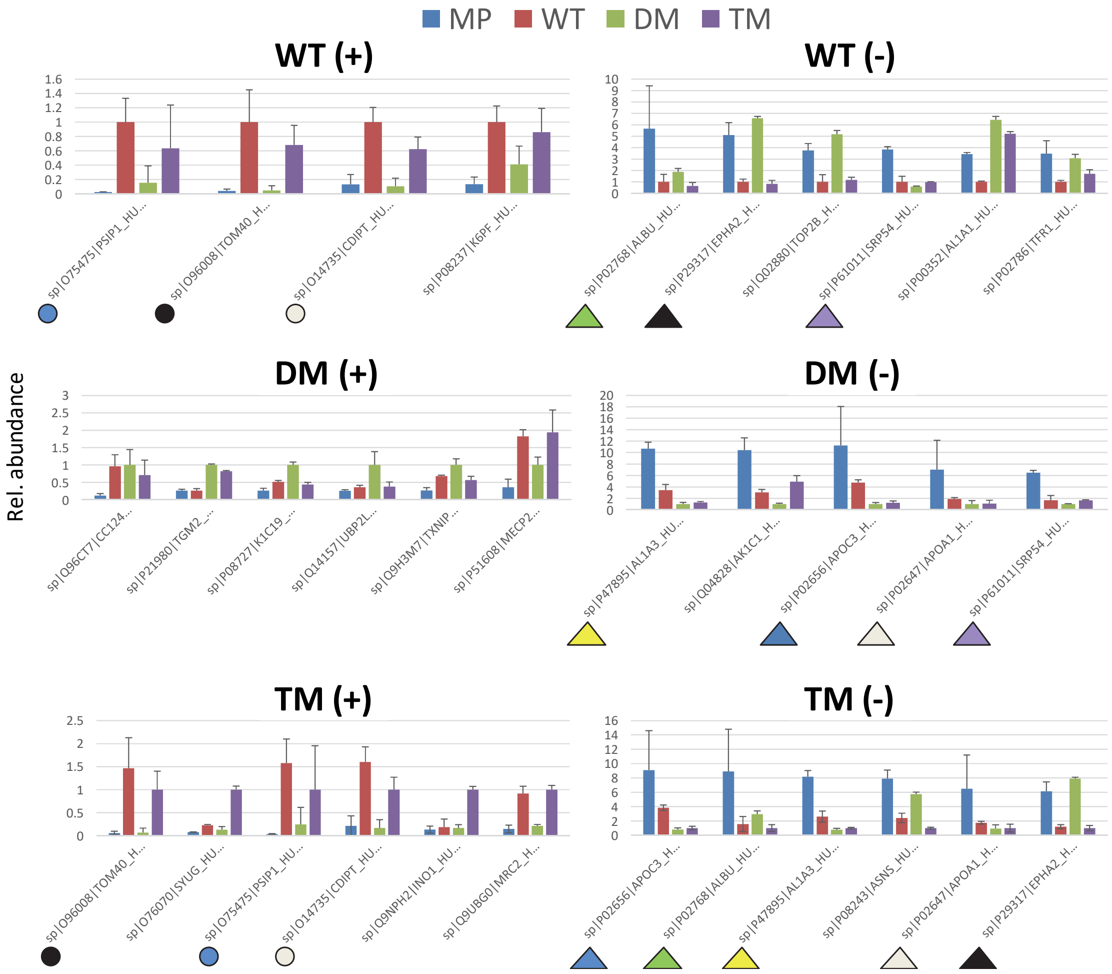
MP WT DM TM
WT (+)
WT (-)
DM (+)
DM (-)
TM (+)
TM (-)
Rel. abundance
sp|O75475|PSIP1_HU...
sp|O96008|TOM40_H...
sp|O14735|CDIPT_HU...
sp|P08237|K6PF_HU...
sp|P02768|ALBU_HU...
sp|P29317|EPHA2_H...
sp|Q02880|TOP2B_H...
sp|P61011|SRP54_HU...
sp|P00352|AL1A1_HU...
sp|P02786|TFR1_HU...
sp|Q96CT7|CC124...
sp|P21980|TGM2_...
sp|P08727|K1C19_...
sp|Q14157|UBP2L...
sp|Q9H3M7|TXNIP...
sp|P51608|MECP2...
sp|P47895|AL1A3_HU...
sp|Q04828|AK1C1_H...
sp|P02656|APOC3_H...
sp|P02647|APOA1_H...
sp|P61011|SRP54_HU...
sp|O96008|TOM40_H...
sp|O76070|SYUG_HU...
sp|O75475|PSIP1_HU...
sp|O14735|CDIPT_HU...
sp|Q9NPH2|INO1_HU...
sp|Q9UBG0|MRC2_H...
sp|P02656|APOC3_H...
sp|P02768|ALBU_HU...
sp|P47895|AL1A3_HU...
sp|P08243|ASNS_HU...
sp|P02647|APOA1_H...
sp|P29317|EPHA2_H...

Fig. S2.

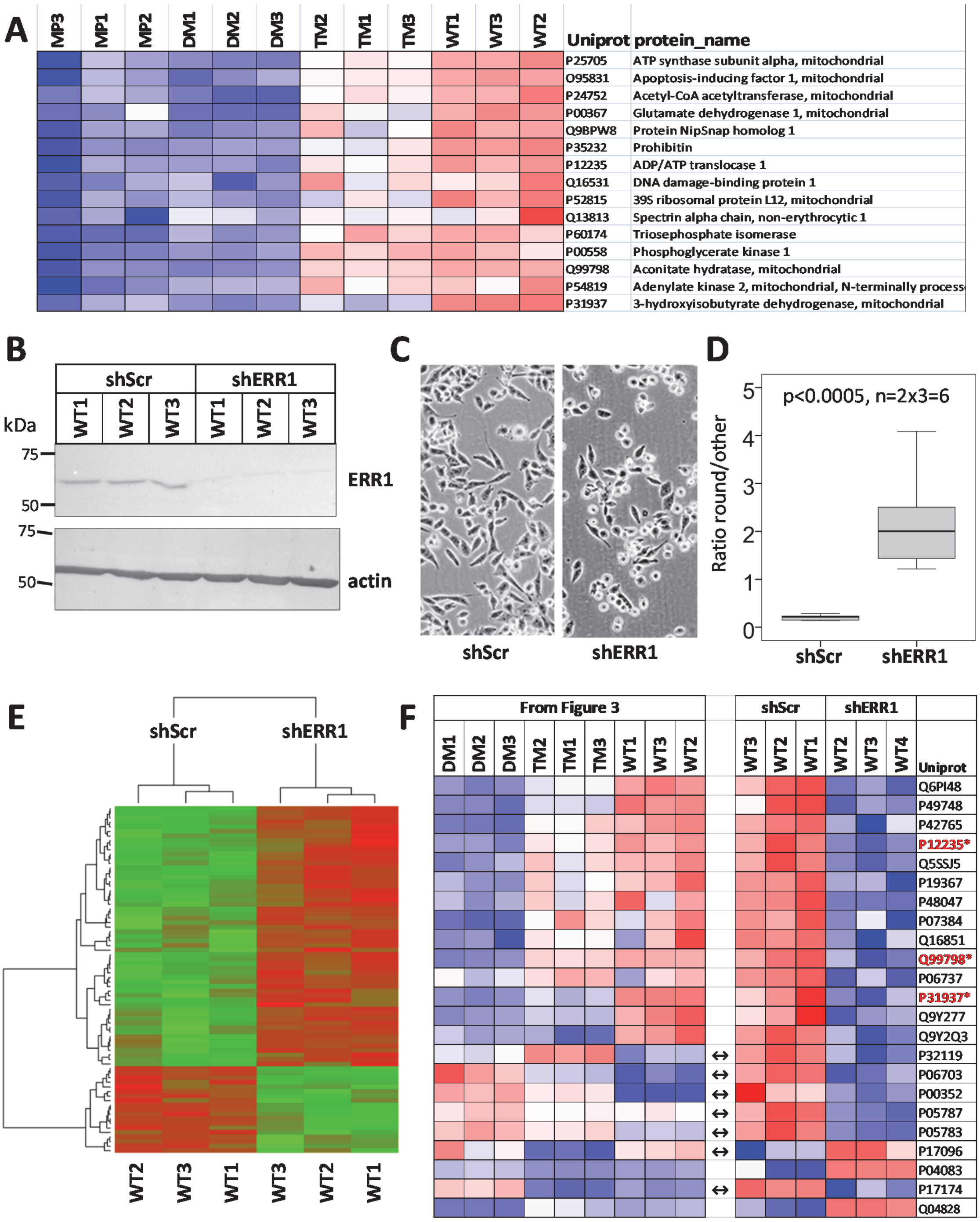
A
MP3
MP1
MP2
DM1
DM2
DM3
TM2
TM1
TM3
WT1
WT3
WT2
Uniprot protein_name
P25705 ATP synthase subunit alpha, mitochondrial
O95831 Apoptosis-inducing factor 1, mitochondrial
P24752 Acetyl-CoA acetyltransferase, mitochondrial
P00367 Glutamate dehydrogenase 1, mitochondrial
Q9BPW8 Protein NipSnap homolog 1
P35232 Prohibitin
P12235 ADP/ATP translocase 1
Q16531 DNA damage-binding protein 1
P52815 39S ribosomal protein L12, mitochondrial
Q13813 Spectrin alpha chain, non-erythrocytic 1
P60174 Triosephosphate isomerase
P00558 Phosphoglycerate kinase 1
Q99798 Aconitate hydratase, mitochondrial
P54819 Adenylate kinase 2, mitochondrial, N-terminally process
P31937 3-hydroxyisobutyrate dehydrogenase, mitochondrial
B
shScr
shERR1
WT1
WT2
WT3
WT1
WT2
WT3
kDa
75
50
ERR1
75
50
actin
C
shScr
shERR1
D
p<0.0005, n=2x3=6
Ratio round/other
0
1
2
3
4
5
shScr
shERR1
E
shScr
shERR1
WT2
WT3
WT1
WT3
WT2
WT1
F
From Figure 3
shScr
shERR1
DM1
DM2
DM3
TM2
TM1
TM3
WT1
WT3
WT2
WT3
WT2
WT1
WT2
WT3
WT4
Uniprot
Q6PI48
P49748
P42765
P12235*
Q5SSJ5
P19367
P48047
P07384
Q16851
Q99798*
P06737
P31937*
Q9Y277
Q9Y2Q3
P32119
P06703
P00352
P05787
P05783
P17096
P04083
P17174
Q04828

Fig. S3.

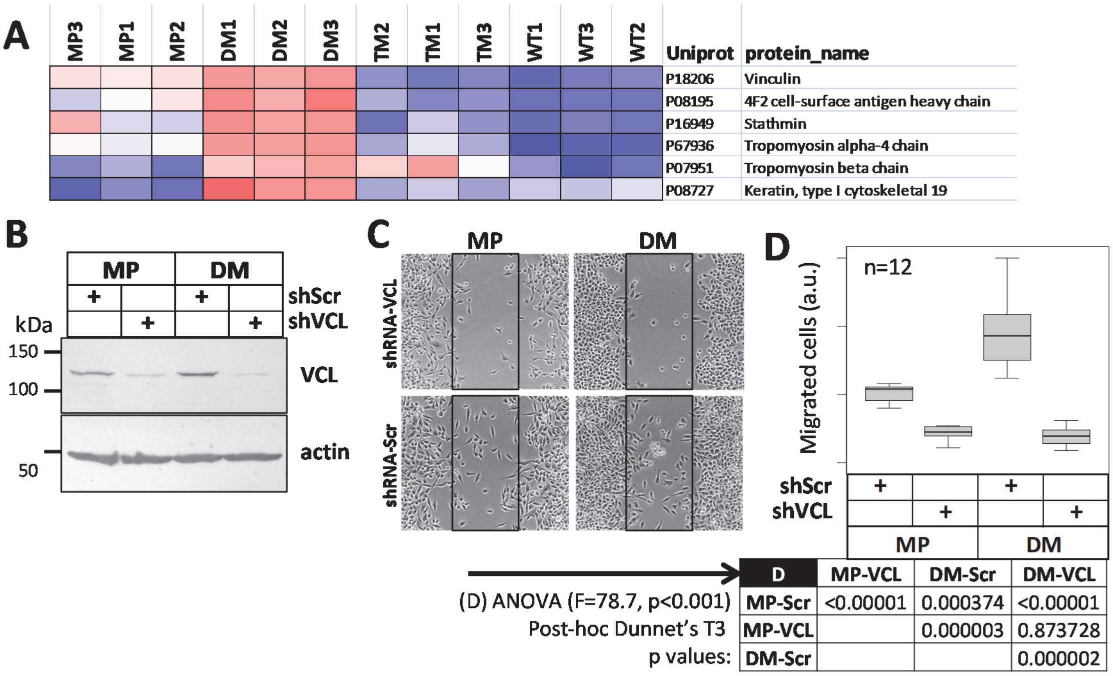

(D) ANOVA (F=78.7, p<0.001)
Post-hoc Dunnet's T3
p values:

D	MP-VCL	DM-Scr	DM-VCL
MP-Scr	<0.00001	0.000374	<0.00001
MP-VCL		0.000003	0.873728
DM-Scr			0.000002

Fig. S4.

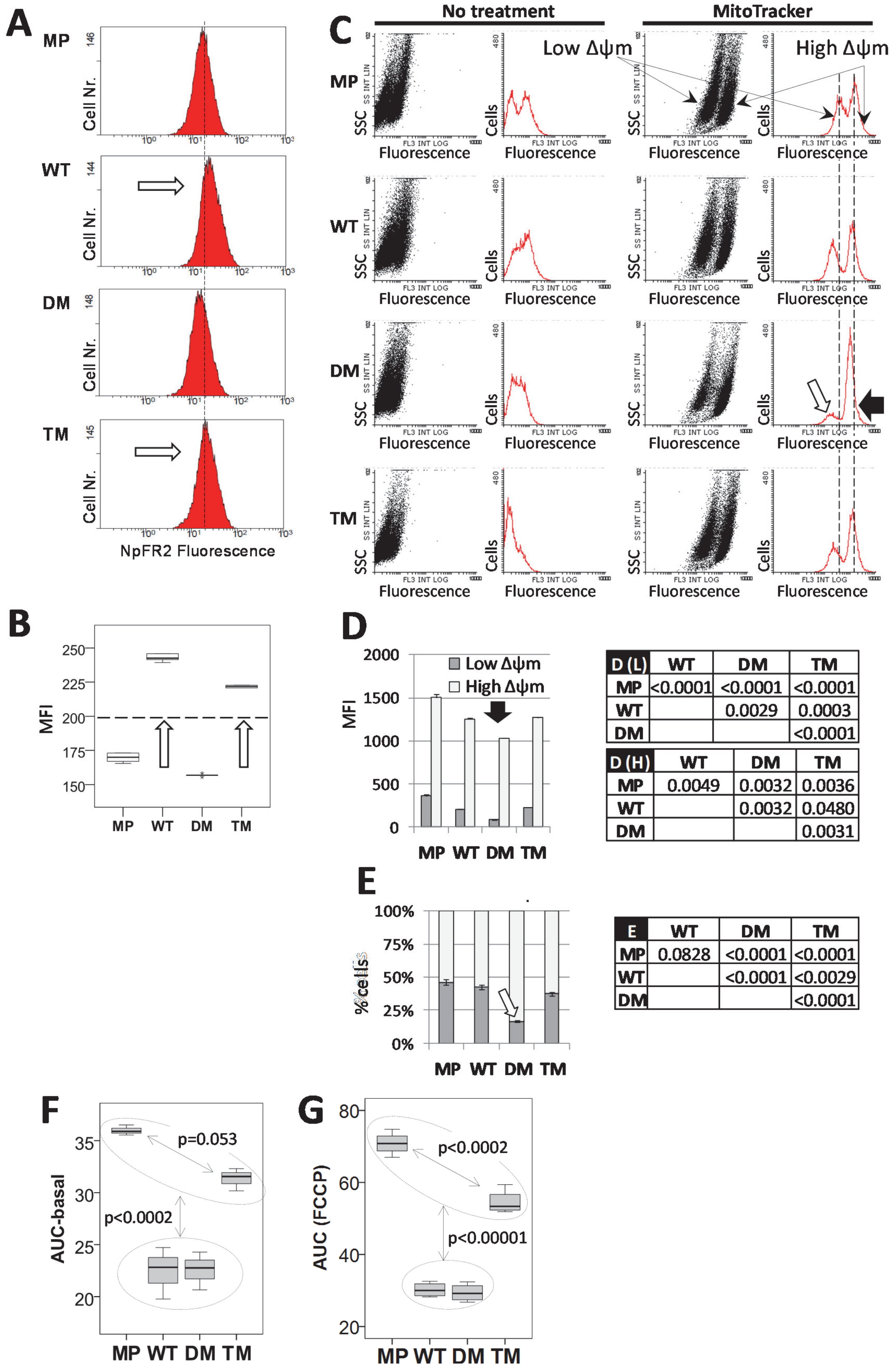
A
MP
WT
DM
TM
Cell Nr.
NpFR2 Fluorescence
C
No treatment
MitoTracker
Low Δψm
High Δψm
SSC
Cells
Fluorescence
FL3 INT LOG
SS INT LIN
B
MFI
250
225
200
175
150
MP WT DM TM
D
2000
1500
1000
500
0
Low Δψm
High Δψm
D (L) | WT | DM | TM
MP | <0.0001 | <0.0001 | <0.0001
WT | | 0.0029 | 0.0003
DM | | | <0.0001
D (H) | WT | DM | TM
MP | 0.0049 | 0.0032 | 0.0036
WT | | 0.0032 | 0.0480
DM | | | 0.0031
E
100%
75%
50%
25%
0%
% cells
E | WT | DM | TM
MP | 0.0828 | <0.0001 | <0.0001
WT | | <0.0001 | <0.0029
DM | | | <0.0001
F
AUC-basal
35
30
25
20
p=0.053
p<0.0002
G
AUC (FCCP)
80
60
40
20
p<0.0002
p<0.00001

Fig. S5.
